# Healing the Separation in High-Conflict Post-divorce Co-parenting

**DOI:** 10.3389/fpsyg.2022.913447

**Published:** 2022-06-17

**Authors:** Alexandra Stolnicu, Jan De Mol, Stephan Hendrick, Justine Gaugue

**Affiliations:** ^1^Department of Clinical Psychology, Faculty of Psychology and Educational Sciences, University of Mons, Mons, Belgium; ^2^Sciences Research Institute, Université Catholique de Louvain, Louvain-la-Neuve, Belgium

**Keywords:** high conflict co-parenting, co-parenting interventions, child’s best interests, divorce, family process, co-parenting, post-divorce co-parenting, parental separation

## Abstract

**Objective:**

Our research aim is to enrich the conceptualization of high conflict post-divorce co-parenting by understanding the dynamic process involved.

**Background:**

The studied phenomena were explored by linking previous scientific knowledge to practice.

**Method:**

We cross-referenced the previous study results with the experiences reported by eight professionals and tried to answer the following research question: how professionals’ experience and previous scientific knowledge contribute to a better understanding of HC post-divorce co-parenting? Individual face to face interviews were conducted and analyzed regarding the qualitative theoretical reasoning of thematic analysis.

**Results:**

Analysis allowed us to highlight how four main axes are related to HC post-divorce co-parenting: (1) Parents for life, (2) Acting in the child’s best interests, (3) Managing disagreements, and (4) Healing the separation.

**Conclusion:**

Our findings capture high conflict post-divorce co-parenting as a multidimensional dynamic process. As such, dealing with co-parenting disagreements must be understood as a moment in a process that is influenced by, and influences, other dimensions.

**Implications:**

Interventions must consider the four dimensions and their reciprocal interactions. The essential elements underlying parents’ difficulties may reside at a multiplicity of levels: inter-relational, contextual, and intrapsychic. Each level contains key potential factors in understanding these families, and in formulating intervention guidelines.

## Introduction

Co-parenting or how parents work together regarding childrearing tasks is an important determinant of the quality of parent–child relationships, positive child adjustment, and the quality of extended family relationships in post-divorce family contexts (Petren et al., 2017). Likewise, co-parenting relationship quality is one of the most powerful factors in explaining families’ psychosocial adjustment after marital breakdown (Pires and Martins, 2021).

Consequently, low interparental conflict is recognized as an important protective factor that enhances children to cope better with their parents’ divorce (Karela and Petrogiannis, 2018). However, maintaining or forging a low-conflict (LC) co-parenting relationship after marital dissolution remains particularly difficult (Darwiche et al., 2021).

Low-conflict (LC) and cooperative co-parenting after marital separation is characterized by the ability of parents to put aside their own conflicts to effectively coordinate the care of their children (Beckmeyer et al., 2014). This type of co-parenting promotes resilience in children (Becher et al., 2019), not only because they benefit from parental cooperation and conflict reduction, but also because of the good mental health and maturity that most likely characterize their parents (Kelly, 2012) which, in turn, may contribute to the quality of parenthood in each household. As Eikrem and Jevne (2022) suggest, cooperative co-parenting requires hard work, even in no-or low-conflict divorces, but the parents do it for their children. In these situations, parents make efforts to (1) continue established parenting practices, (2) shield the children, and (3) deal with their own emotions. Same authors indicate that the inherent premises for successful co-parenting are trust in each other’s ability to take care of the children and their own knowledge of the emotional bonds between the children and each parent. In the same line, previous research (Stolnicu and Hendrick, 2017; Stolnicu, 2020) shows that LC post-divorce co-parenting is a three-dimensional process that is constantly evolving over time. In these cases, parents seem to obey three rules: (1) considering each other as “parents for life,” (2) “acting in the child’s best interests,” and (3) “managing disagreements.” “Parents for life,” involves recognizing the qualities and educational skills of the other parent, the respect for one’s parenting role, the desire to maintain child bond with the other parent as well as the acceptance of the other parent’s parental rights and co-parental responsibility. “Acting in child’s best interests” involves promoting dialogue with children, valuing their well-being, and respecting new parental figures. In order to “Manage disagreements,” parents in LC co-parenting relationships demonstrate empathy, relational balance, as well as transparency and flexibility in arrangements matters concerning their children.

On the other hand, parents in prolonged conflict are closed off from a storyline of cooperative co-parenting. While cooperation requires the willingness to influence and to be influenced by the other, parents in HC situations show no available positions of cooperativeness to take up (Stokkebekk et al., 2021). These parents manifest low levels of life satisfaction, high levels of divorce-related distress, and inconsistent parenting (Lamela et al., 2016). They often see themselves as victims as their experience of HC involves pervasive mistrust (Rød et al., 2013). HC situations are often characterized by recurrent legal disputes, high degree of anger and mistrust, communication problems linked to hostility, arguments, and disappointments, verbal and/or physical aggression, and difficulties in focusing on the needs of children as separate from the needs of the parents (Bacon and McKenzie, 2004; Yárnoz-Yaben and Garmendia, 2016). Consequently, high levels of interparental conflict are extremely detrimental to the development of children (Duerr and Hautzinger, 2019) by engaging negative psychological effects that tend to persist over time (Moral et al., 2021). For professionals working with families who continue to quarrel after marital separation, it is one of the most complicated areas of their practice. Ongoing legal battles, or the threat of new legal proceedings, with the stress and financial consequences that this imposes, make these interventions even more difficult (Van Lawick and Visser, 2015). This lack of conceptual clarity is considered to be a major source of confusion for both researchers and professionals (Anderson et al., 2011). Despite the abundant literature, there is a lack of concise and contemporary definition of HC post-divorce co-parenting (Francia et al., 2019). The term “high conflict” (HC), which continues to have a lot of meaning in family law, refers to large variety of situations that overlap to some extent, but which are in many ways quite different (Smyth and Moloney, 2019).

Therefore, HC parental separations are a major challenge for child welfare professionals as well as for the justice system. Sometimes children in HC co-parenting situations may not get the help they need because of the lack of information and training for professionals on this phenomenon (Houston et al., 2017).

Considering these challenges, the present study aims to enhance the conceptualization of HC post-divorce co-parenting. Through our research design we hope to deepen the understanding of HC co-parenting situations in terms of behaviors and interactions between parents, but also between parents, children, and other family members.

## Materials and Methods

### Rationale

Our research aim is to approach HC post-divorce co-parenting by understanding the dynamic process involved. LC co-parenting and HC co-parenting are very different, however in both situations, divorced parents deal with important difficulties. Thus, cooperative co-parenting does not mean the absence of obstacles. Rather, maintaining this type of relationship after parental separation requires hardwork, even in no- or LC divorces, but parents do it for their children (Eikrem and Jevne, 2022). Research shows that parents make efforts to obey the three LC co-parenting rules: (1) considering each other as “parents for life,” (2) “acting in the child’s best interests,” and (3) “managing disagreements” (Stolnicu and Hendrick, 2017; Stolnicu, 2020). Despite countless difficulties, parents in LC situations seem to obey these guidelines naturalness. Therefore, it appears important to better understand the specific complexities that hinder parents in HC situations from observing these principles. To this end, professionals’ experience is a valuable resource which provides access to a variety of experiences regarding the studied phenomena.

This article is the third part of a larger research project.

#### Previous Research

Firstly, Stolnicu and Hendrick (2017) examined the experiences of four participants from two heterosexual parental couples who consider themselves as having successfully negotiated marital breakdown. Phenomenological interpretive analysis (Smith et al., 2009) allowed to identify three key ideas that govern LC post-separation co-parenting behaviors. Therefore, LC co-parenting is conceptualized as an interactional three-dimensional process that is constantly evolving over time. Consequently, in LC co-parenting situations, parents seem to obey three rules: “Parents for life,” “Acting in the child’s best interests,” and “Managing disagreements” (Stolnicu and Hendrick, 2017; Stolnicu, 2020).

Secondly, Stolnicu (2020) replicated this study with a larger population: 12 participants from six heterosexual parental couples. Same results were obtained. LC post-divorce co-parenting relationship was defined as a tridimensional process based on the interaction of the above three dimensions.

#### Present Study

The present study focuses on studying HC post-divorce co-parenting by crossing previous knowledge (Stolnicu and Hendrick, 2017; Stolnicu, 2020) with professionals’ experiences regrading interventions in HC post-divorce co-parenting situations.

By trying to answer the following question: how professionals’ experience and previous scientific knowledge (Stolnicu and Hendrick, 2017; Stolnicu, 2020) may contribute to a better understanding of HC post-divorce co-parenting? Our objective is to deepen the studied phenomena by linking previous research results to practice. From this perspective, we chose a qualitative theoretical reasoning of thematic analysis (Braun and Clarke, 2006). This approach complemented the research question. Theoretical coding allowed us to deepen previous results regarding post-divorce LC co-parenting and further develop the context of HC co-parenting. As such, theoretical knowledge preceded data interpretation, and a preliminary framework was applied to the data.

Table 1 summarizes previous study results (Stolnicu and Hendrick, 2017; Stolnicu, 2020) regarding LC post-divorce co-parenting.

**Table 1:** Results of Stolnicu and Hendrick (2017) and Stolnicu (2020).

S.no	Main themes	Sub-themes
1.	Parents for life	1.1 Recognize the other parent’s qualities and skills 1.2 Respect the other’s role as parent and support the parent–child relationship 1.3 Acknowledge parental rights and parental responsibility according to inter-parental trust
2.	Acting in the child’s best interests	2.1 Treasure the child’s well-being 2.2 Encourage and support dialogue and focus on the children’s experiences 2.3 Respect new parental figures
3.	Managing disagreements	3.1 Accept that conflicts are inevitable but manageable 3.2 Maintain the relational balance 3.3 Maintain flexibility and stability

Therefore, in accordance with Braun and Clarke (2006) theoretical approach, we were interested in the way that the themes cited above (see Table 1) play out across the data and we focused on that particular feature in coding the data. Analysis may result in a number of themes around studied phenomena, which may include, speak to, or expand on something approximating previous studies’ original themes.

In line with our objective of relating previous scientific knowledge to practice, we chose a collaborative approach to collect the data by involving our participants as full agents in the process of knowledge production. Consequently, participants were informed of the objective and themes of our research and received the related interview guide. The collaborative approach permitted participants to take a metaposition and therefore enabled them to have a broader view regarding possible links between their own professional practice and the proposed theoretical knowledge of the studied phenomena.

The four authors of this study are clinical psychologists. Interviews were carried out and analyzed by the first author.

### Participants

To participate in the study, professionals had to have a consistent experience in working with families experiencing HC separation. They must also have completed additional training in systemic psychotherapy and/or family mediation in order to have the theoretical-clinical knowledge necessary for understanding HC co-parenting situations.

Participants were contacted through clinicians’ professional networks. Eight professionals were selected. At the time of the interviews, the participants had between 4 and 20 years of professional practice. The participants were: a clinical psychologist trained in family psychotherapy, a clinical psychologist trained in family mediation, two social workers trained in family mediation, a lawyer trained in family mediation, and three psychiatrists trained in family psychotherapy. It should be noted that our interviewed practitioners had different professions and approaches to interventions in HC co-parenting situations. This allowed us to reach an interdisciplinary understanding of the studied phenomenon. All the participants signed an informed consent form.

### Procedure

First, we identified experts according to the inclusion criteria. Subsequently, and in accordance with our collaborative approach, we sent the participants the necessary documents: our previous article (Stolnicu and Hendrick, 2017; Stolnicu, 2020) as well as the interview guide. The guide informed them of the procedure and was intended to allow them to give targeted feedback of their own experience. Face to face individual interviews were carried out on average 2 weeks after receipt of the documents. The average duration of the interviews was 1 h 30 and they were carried out at the workplace of each participant.

### Data Collection

Semi-structured interviews were carried out according to the guidelines provided by Braun and Clarke (2006). Two main issues were discussed with each participant. Thus, based on their professional experience, the practitioners were asked about the possibility of:

1. Linking, where appropriate, the different main themes and related themes identified in the research (Stolnicu and Hendrick, 2017; Stolnicu, 2020) during their professional practice regarding HC co-parenting situations.

Example questions: “According to your professional experience, how might the sub-theme of recognizing the other parent’s qualities and skills be understood in HC post-divorce co-parenting situations? Do parents recognize the other parent’s qualities? How does recognizing the other parent’s qualities manifest in HC co-parenting situations?”

2. Completing these results with respect to their professional practice, for example by suggesting one or more new main themes or related sub-themes.

In line with our qualitative approach, the interview guide was used in a flexible way in order to allow the emergence of new themes. We investigated the two abovementioned points, while encouraging our participants to develop and clarify their answers.

Interviews were audio recorded and transcribed verbatim.

### Data Analysis

Analysis of the interviews was carried out using the theoretical thematic analysis method (Braun and Clarke, 2006). The six stages of the analysis were: (1) Becoming familiar with the data. (2) Generating initial codes—Codes identify a characteristic of the data that seems interesting in the sense of providing insight into the studied phenomenon. (3) Searching for themes—in line with the theoretical approach of the thematic analysis this stage involves linking potential themes with pre-existing notions. Thus, for each pre-existing theme, the researcher tries to gather the potential themes linked to it with the corresponding verbatim extracts. The researcher must keep an open mind to the possibility of the emergence of new themes relevant to the research question. Thus, potential themes that do not relate to pre-existing themes can form one or more new themes. (4) Reviewing themes—this stage involves two levels of review: checking the relevance of the themes in relation to the coded extracts (level 1) and, in relation to all the data (level 2). (5) Defining and naming themes, and (6) Producing a report—the report should tell the “complicated story of the data” in a way that convinces the reader of the validity and merit of the analysis. It is an analytical narrative illustrated with data extracts that provide arguments in relation to the research question.

## Results

Based on their professional practice, the participants identified the previous studies’ (Stolnicu and Hendrick, 2017; Stolnicu, 2020) three main themes (Table 1) as part of their own clinical experience. These three main themes, and the nine related sub-themes, were recognized but mainly considering the complications involved in HC post-divorce co-parenting situations. In other words, professionals find the three axes descriptive of HC post-divorce co-parenting and identified for each dimension specific difficulties that impede co-parenting evolution toward a LC relationship. The practitioners were able to illustrate these themes with examples of clinical situations from their own practice.

Moreover, a new main theme was added: (4) Healing the separation.

Table 2 summarizes the results regarding the fourth theme “Healing the separation” that was added in this study.

**Table 2:** The fourth main theme added in this study.

S.no	Main theme	Sub-themes
1.	Healing the separation	Evacuate the “*emotional overflow*” Identify “*what is blocking*” and work it out Distinguishing the conjugal past from the co-parenting present

For each main theme and related sub-theme, specific characteristics of HC post-divorce co-parenting were added. The results section focuses on the presentation of the new aspects and aggravating factors highlighted in this study. The professionals also drew attention to some of the difficulties associated with working with these families. The following section will provide, for each main theme, a brief summary of our results.

### “Parents for Life” in HC Post-divorce Co-parenting

Most professionals agreed that “parents for life” was descriptive of HC co-parenting. However, negative labeling of the other parent and wounds related to the conjugal conflict may shape a distorted view of the ex-spouse as a parent.

Negative labeling or/and a negative “compacted” representation of the former spouse may impede parents in recognizing the other parent’s parenting qualities and skills. In certain situations, a parent may be mainly characterized, by the other parent, with respect to an isolated event, concerning a behavior considered as negative and interpreted separately from its contextual backdrop. Parents may also find it difficult to separate wounds related to the conjugal conflict from the ex-spouse’s image as a parent. Therefore, the other parent’s potential parenting skills are confused with a negative representation of this parent as an ex-spouse.

“Seeing beyond an event that may have been experienced as a trigger, or as difficult because the parent was not recognized as competent at that time, how can we also see that this parent is not reduced to that one event?” (…)“the other parent is demonized as a person and as a parent.” (Professional G).

Practitioners explained that frequently in HC situations the other parent’s role is not respected and their relationship with the child is hindered. Parents may send conflicting messages to children. Thus, a parent may verbally encourage the child to maintain a relationship with the other parent. Nevertheless, the child may actually feel that any act that responds to this demand could be experienced as unbearable by the same parent who encouraged them to do so. Additionally, the child’s refusal to spend time with the other parent may be claimed as a reason to discourage the child’s relationship with that parent.

“Sometime what is said explicitly is ‘yes, yes, it is fundamental that you keep a link with your dad’ and then in practice, as soon as this happens a little bit, it is unbearable” (Professional F).

“Mom refused to let the child go to his Dad. Because this little 4-year-old boy told his Mom…‘I do not want to go to daddy’s’” (Professional A).

Some parents may be reluctant to ask for, and consider, each other’s opinions about decisions concerning their children. Therefore, by considering themselves individually as the only ones able to make decisions about the children, they may choose to put the other parent “aside.” The lack of inter-parental confidence related to former ex-spouses’ relational wounds, may also hamper the acknowledgement of parental rights and parental responsibility.

“There is no inter-parental trust and/or for many reasons, one parent feels that they have all the rights in the end.” (Professional F)

“There are a whole series of wounds that require healing or time before they can say, “Despite that, I can trust him as a parent, and he is still a parent.”” (Professional D).

### “Acting in the Child’s Best Interests” in HC Post-divorce Co-parenting

Interviewed practitioners approved that “acting in the child’s best interests” is illustrative of HC co-parenting. Yet, the valuation of the child’s well-being can be obstructed by the suffering associated with marital separation, as well as by the adjacent conflicts.

Sometimes, child’s best interests are “twisted.” For instance, professional F explained that in certain situations one parent can explicitly encourage the child to reconnect with the other parent. However, the child may feel, implicitly, that if they respond to this request they will suffer “terrible relational consequences.” The relational context is “perverting the bond.” The parent can therefore say that they are acting in the best interests of the child, but their behavior reveals their own needs related to their own wounds caused by the marital separation. These observations may also be linked to one of the comments mentioned above, the preservation of the parent–child relationship. Moreover, parents’ feelings of betrayal and vengeance may outweigh children’s best interests. Thus, parents manage to “forget” the children. For these parents, the past wounds take precedence over the way they represent the well-being of their offspring.

“They justify always acting in their child’s best interests, but it is not an objective interest, sometimes the child’s best interests are twisted in the conflict.” (Professional D)

“The sense of revenge and betrayal are such that the child’s best interests become secondary” (Professional H)

The practitioners confirmed the need for parent–child dialogue. Nonetheless, they stressed some difficulties. Thus, the parental practice of encouraging and supporting dialogue and taking an interest in the children’s lives can be considered as a “double-edged sword.” Sometimes, parents can motivate the emergence and persistence of inter-parental conflict through their children’s complaints. Professional D highlighted the exploitation of the children’s words, which are exaggerated and “systematically used against the other parent.” Rather than taking a real interest in the child’s experience, parents can encourage and orient dialogue with the child in order to involve them in the inter-parental conflict.

“I do not feel like that they are really asking them how they feel, rather they are trying to get them into the conflict and practically asking them to take sides” (Professional E)

Nevertheless, the practitioners stressed possible misinterpretations due to a lack of parent–child dialogue. Despite the difficulties mentioned above, the practitioners stressed the importance of parent–child dialogue. Sometimes children’s interpretations of parental decisions and behaviors are influenced by inter-parental conflict. Taking an interest in children’s experiences can avoid possible misunderstandings. Otherwise, misinterpretation of parental attitudes may become a source of suffering and conflict in the parent–child relationship. Issues related to money and sharing of economic responsibilities within the co-parenting relationship were identified as particularly sensitive.

“We hear children saying, ‘Do you remember the thing with the school trip, he did not pay for it, yet it was really important to me.’” (…) “Sometimes the child may understand this as a lack of attention, when in reality the parent does not have any money.” (Professional F).

The practitioners also supported the distinction between encouraging the child to express their experience and granting them decision-making power. Furthermore, professional F sees giving decision-making power to children as “too heavy a burden to bear” and a source of insecurity. As a result, a child’s words should not be used “either in the inter-parental conflict or against the child,” nor as if the child “had all the power.” It is essential to “act in their best interests,” even if their best interests are different from what the child says they want. Thus, if a child says they no longer want to see one of their parents, it is necessary to “understand their reasons and to reflect on various hypotheses related to this request.” Consequently, the importance of encouraging children to express their experiences, and to help parents to give proper value to their children’s words was highlighted.

Regarding the new parental figures, professional H explained that sometimes, due to the intensity of inter-parental conflict, “children do not feel entitled to value stepparents.” The unwieldly nature of the family context and the prohibition to value stepparents makes children have to face significant loyalty conflicts.

Professional E stressed the lack of sufficient knowledge of the other parent’s new spouse as a supplementary difficulty impeding their acceptance as a parental figure. This may disable the possibility of “building relationships based on trust” and cooperation between parents and stepparents. However, indirectly, children are often entrusted to their “unknown stepparents.”

Sometimes, parents may perceive the other parent’s new spouse as a threat, as a rival who seeks to take their place regarding the child. Moreover, in a family context of rivalry and confusion, stepparents are rarely recognized and often criticized. Our findings highlight the importance of supporting inter-parental cooperation and legitimizing stepparents as parental figures.

“We often hear that the former spouse says ‘he is not going to take my place as a father anyway’(…)‘I’m the father’, but it is this parent who has to, and this also takes time, who has to give permission little by little, and say ‘There, you can take on a parental role’” (Professional G).

### “Managing Disagreements” in HC Post-divorce Co-parenting

Professionals admitted that “managing disagreements” is explanatory of HC co-parenting. Still, parents in HC situations show a diminished forbearance for unexpected changes and considerable difficulties during negotiation. Disagreement and interparental conflict may rapidly re-emerge through these incidents and appear to be experienced as unmanageable.

A common problem arises from parents’ negative misinterpretations of each other’s actions. Practitioners’ attempts to highlight one parent’s “positive” parenting behaviors can prompt the other parent’s skeptical explanations. In a relational context characterized by a lack of communication, one parent’s apparently non-confrontational behavior is interpreted in compliance with what has already been deemed acceptable within their conflictual relationship.

“I said, ‘Yes, but the father did keep his word, etc.,’ but she always replied, ‘Yes, but I’m sure it was for this or that’” (Professional D).

Additionally, the impatience and the “naivety” of some parents with respect to inter-parent conflict resolution were highlighted. Some parents think that a judge will immediately put an end to the conflict; therefore, parents may take action through the legal system based on erroneous premises.

“Often, the parents who come to see us want an immediate, direct solution. That’s it. And that is what they may also think when going to court… ‘The judge will decide, and after that everything will be fine’…well, no. It does not work that way.” (Professional B).

Moreover, professional H explains that “in order to avoid conflict,” parents can “actively hide relevant information” concerning their children. In their reasoning, conflicts must be avoided because they are unmanageable.

Disagreement reasons, expressed explicitly by parents, may conceal feelings of injustice related to the conjugal relationship and marital separation. The implicit motives of the conflict may be related to a ledger of accounts, to debt payment regarding narcissistic level reparations. Thus, implicit motivations may strongly differ from the explicitly stated reasons.

“There is a lot of spite, something which has not been dealt with. As if parents have a debt to pay. Parents are so caught up in this war that they do not know how to move on and think about their child.” (Professional B).

In some cases of HC co-parenting, child’s interests are reduced to a story of two-against-one coalitions with one parent winning and the other losing.

“The issue is not ‘what are we really thinking, what is the best school for the child?’ but rather ‘Who will win this time?’” (Professional F).

In most HC situations, flexibility and stability are contradictory. Some parents think that only inflexibility, manifested through unconditional respect of the pre-established decisions, may allow stability to be reached by avoiding inter-parental disputes.

“So for them to have stability, there cannot be flexibility.”…“They prefer inflexible things, rather than entering into a dialogue”(…) “Because it avoids disputes” (Professional E).

Sometimes, their own separation distress disables parents from spontaneously seeing the benefits of inter-parental flexibility for their children. Pain and emotions associated with separation may lead parents to dyadic functioning. The child’s experience does not seem to be considered. In this context, inflexibility becomes a form of inter-parental vengeance for endured suffering. Professional B explains that “conflict allows parents to evacuate their emotional overflow.”

### “Healing the Separation” in HC Post-divorce Co-parenting

In order to complete the conceptualization of HC post-divorce co-parenting, the professionals felt it necessary to add a new dimension “Healing the separation.” The four axes are interconnected in the understanding of HC co-parenting. As a result, some aspects of this fourth theme have already been highlighted in the identification of the other axes mentioned above. Practitioners’ vigilance in healing the separation was considered as an obligatory condition enabling intervention focused on helping parents regarding the other three dimensions. The professionals’ comments regarding this fourth main theme are mostly focused on intervention guidelines aiming to help parents through their process of “Healing the separation.”

In HC situations, parents’ “emotional overflow” may prevent them from trying to improve their co-parenting relationship. The practitioners stressed the importance of soothing parents’ suffering, their emotional wounds and anger. Parents’ suffering may be related to feelings of injustice and need for compensation, which in some cases may take the form of vengeance. Therefore, practitioners must help parents evacuate their real suffering in order to prepare the field for more focused co-parenting interventions.

“If we cannot listen to them about their suffering, we’ll never be able to move on” (…) “we have to help them overcome this…not this spirit of revenge, but somehow it’s a little bit of that.”(…) “It’s something like … ‘If you do not let me talk about it and tell you how much I’ve been hurt, I will not be able to move on to something else!’” (Professional A).

Practitioners must respect the parents’ rhythm and their need to express their experience of the separation. Interventions focused on co-parenting may be hampered by practitioners’ desire to “look forward,” particularly when it does not respect the parents’ timing related to their specific needs.

“With couples in the middle of a crisis, if you do it too early, they do not hear it. They’re not ready enough, so there is a time for them to be able to externalize their overflow and then be able to move forward and hear it” (Professional B).

Acting this way may promote a better understanding of the conflictual relationship specific to each family and enables parents to identify the aspects that continue to trap them in the conflict.

Professional F highlighted that the influence of “each parent’s identity constructed within the conjugal relationship” may block parents into co-parenting conflict. For example, if, within the conjugal relationship, one of the parents built themself “an identity as a victim of a narcissistic pervert,” teaming up with that ex-spouse in terms of co-parenting, may be experienced by the “victim” parent as “unbearable.” Any possible collaboration may be blocked by this identity dimension. In these cases, preliminary individual sessions with each parent are necessary. Children’s difficulties to “grow” within a family context considered to be extremely threatening by one of the parents were also highlighted. As mentioned above, the “victim” parent may surround the child with enormous precautions regarding the “terrible danger” represented by the other parent labeled as a “narcissistic pervert.”

Professional F explains that “for some people, staying in the fight is them not falling apart.” Consequently, in order to ensure one’s own psychological survival, parents may push inter-parental conflict to extreme and destructive dimensions. Therefore, factors related to each parent’s psychic economy may block co-parenting collaboration.

Financial aspects are often the cause of inter-parent conflicts. For instance, professional H states that “even though parents may be able to reach agreements concerning other aspects of co-parenting” economic organization and “sharing financial responsibilities” may remain an “major source of conflict.”

Co-parenting relationships may also be hampered by each parent’s difficulties linked to their own past family experience. Not feeling recognized by the other parent concerning suffering related to past suffering may prevent parents from responding positively to co-parenting focused interventions. Therefore, reciprocal inter-parental recognition of past wounds, and legitimization of adjacent suffering, may be experienced by parents as a form of healing. This could break the deadlock that holds parents in a conflict and prevents co-parenting improvement. Therefore, recognition of suffering is an essential condition allowing the transformation of conflicts and the evolution of inter-parental relationships.

“Thankfully, transformation is sometimes possible, but for it to be possible, there really has to be this recognition of suffering for them to move on.” (Professional G).

Practitioners stressed the timing of grief regarding separation. Indeed, parents may find themselves at different stages of the acceptance of marital breakdown. Therefore, remaining in conflict can be understood as a way of maintaining contact.

“There is a grieving of the relationship that it still left to be done by one parent but not by the other. And I think time is important.” (Professional E).

Difficulties in adapting to changing functions within the new family dynamics, may also block parents in conflict. Professional F explains that it is “almost a question of territory” if, before separation, one parent managed most of the parenting issues, it could be complicated for that parent to give up “something of their functions.” As a result, in the post-separation family context, if the other parent is required to become more involved in parenthood, their attempts to perform these functions may become a source of conflict.

The conjugal past may influence conflicts within the co-parenting relationship. For example, professional F explains that sometimes, “the conjugal past may completely penetrate” co-parenting, and therefore “conceal” the other three dimensions, discussed above.

Additionally, professional G explains that when former wounds keep influencing co-parenting, parents may “interpret” each other’s behavior within the “former background” of their conjugal past.

“Forgiveness” may lead to improvements within the co-parenting relationship. In order to regain a certain “serenity,” some parents have consciously chosen to forgive and free themselves from “hate.” Parents’ ability to “remain child-focused” and therefore “cope with conflict and deal with it serenely” may be also linked to their ability to forgive. The questioned practitioners emphasized the necessity to distinguish the conjugal past from how much this past impacts the present. In other words, a very painful past “may or may not” affect co-parenting. As such, it is important for consultants to consider each person’s subjective experience in order to understand how “the past and psychic identity” may affect “the ability to team up” within the co-parenting relationship after the marital breakdown.

“How all of this, the past and the psychological construction act on the ability to … actually act today regarding the ability to team up. It’s not just the past, it’s how we actually live it now. Because this past can be very painful. I think of a mother who said to me ‘I have truly forgiven’, that the past was very painful but now she is in a situation of appeasement. It’s about ‘How this past is still active today in the background?’” (Professional F).

## Discussion

This paper aims to enhance HC post-divorce co-parenting conceptualization by linking previous knowledge (Stolnicu and Hendrick, 2017; Stolnicu, 2020) to professionals’ experience. Our findings capture HC post-divorce co-parenting as a multidimensional dynamic process involving four main axis: (1) “Parents for life,” (2) “Acting in the child’s best interests,” (3) “Managing disagreements,” and (4) “Healing the separation.”

In HC post-divorce situations, professionals find these axes illustrative of HC post-divorce co-parenting and identified for each dimension specific difficulties that impede co-parenting evolution toward a LC relationship.

For instance, these parents often have significant difficulties in recognizing the skills and qualities of the other parent and find it difficult to separate the wounds linked to the marital conflict from the image of the ex-spouse as a parental figure. Thus, the possible parenting skills of the other parent are confused with the negative image of the same person as a former spouse. In these cases, the other parent is demonized as a person and as a parent. Likewise, Disner et al. (2011), three forms of bias may be noticed: selective perception, selective memorization, and biased cognitive processing. In other words, a parent can perceive their ex-spouse in a distorted way, so that they only grasp the information which confirms their negative prejudices and that they, on the other hand, become “blind” regarding the information which does not conform to their prejudices.

If in LC co-parenting (Stolnicu and Hendrick, 2017; Stolnicu, 2020) the place of the other parent is respected and their link with the child is encouraged, in situations of HC co-parenting our results indicate the opposite. Indeed, the place of the other parent is not respected and their bond with the child is hampered.

Interparental dialogue HC co-parenting situations are characterized by a fundamental lack of trust, each parent being constantly attentive to the possible hidden motives of the other parent (Gulbrandsen et al., 2017). Our analysis confirms and supplements these observations by adding that in situations of HC co-parenting post marital separation, the lack of interparental trust seems to play a primordial role in parents’ difficulties to admit reciprocal parental rights. Therefore, HC co-parenting is opposite to LC co-parenting (Stolnicu and Hendrick, 2017; Stolnicu, 2020) where the trust gained before the divorce allows parents to continue their parental duties and accept shared parental responsibility.

In HC co-parenting situations, parents experience significant difficulties in acting in the child’s best interests. The valuation of the child’s well-being can be hampered by the suffering associated with marital separation, as well as by the conflicts which run parallel to this difficult transition. This prevents parents from taking the necessary distance to ensure the well-being of their children. In addition, when faced with strong feelings of revenge and betrayal, parents are no longer able to act in the best interests of their child who becomes secondary and may also be exploited in conflict. So, a parent may say that they are acting in the best interests of their child, but in reality, their behavior responds to their own needs related to the wounds of marital separation. Similarly, people who feel irreparably hurt by marital separation may try to protect themselves psychologically by transforming feelings of vulnerability into powerful feelings of injustice accompanied by grievances and desires to receive justifications and compensation for the damage inflicted by the other parent (Demby, 2017).

Our results specify that in some cases of HC co-parenting, child’s interests are reduced to a story of two-against-one coalitions with one parent winning and the other losing. Indeed, parental narcissism and empathy can influence the quality of the co-parenting relationship of separated parents (Baum and Shnit, 2003; Donner, 2006). Therefore, in HC situations, parents are so caught up in their conflict that they are no longer fully aware of the well-being of their children (Van Lawick and Visser, 2015).

Even if the need for parent–child dialogue, highlighted by Stolnicu and Hendrick (2017) and Stolnicu (2020) was confirmed by the professionals interviewed in our study, certain difficulties specific to situations of HC co-parenting were stressed. Therefore, the parental practice of supporting dialogue and taking an interest in the experiences of children can be seen as a double-edged sword. Several obstacles were suggested. For example, parents may interpret their child’s needs according to their own needs, and the child’s speech may be influenced by their loyalty to their parents. Likewise, when exposed to high levels of interparental conflict, children feel that they cannot get closer to one of the parents without being disloyal to the other (Brown et al., 2009). Physical and psychological symptoms may also be noticed when children experience high levels of stress related to loyalty conflicts (Bernet et al., 2016).

Despite the difficulties mentioned above, our study stresses the importance of parent–child dialogue. Sometimes the child’s interpretations of parenting decisions and behaviors are influenced by interparental conflict. Therefore, being interested in the experiences of children can avoid possible misunderstandings. On the contrary, the distorted interpretations of parental attitudes may become a source of suffering and conflict in the child–parent relationship. In line with Taylor (2001), our results objectify the idea that children can feel responsible for their parents’ divorce and that it is important that they be able to express their experiences and their real needs in relation to this event. We thus agree with Afifi et al. (2006) on the need for a dialogue between parents and children around parental separation in order to facilitate the necessary adaptation.

In the same line with Bullard et al. (2010), our results objective the ongoing difficulties related to the parenting role of stepparents. In HC co-parenting situations, the relational context is often characterized by rivalry and confusion regarding the roles of different parental figures which parents in a HC co-parenting show a very low tolerance for unforeseen changes and significant difficulties during negotiation. For these parents, disagreement and interparental conflict quickly resurfaced during these incidents and seemed to be experienced as unmanageable. The anger and feelings of injustice associated with marital separation can hijack parents’ ability to negotiate within the co-parenting relationship (Cohen and Finzi-Dottan, 2012).

Sometimes the apprehension of disputes makes parents in HC situation avoid dialogue. These parents can actively decide not to exchange all information about the children, in order to avoid conflicts. In the long term, this attitude risks blocking the parents because, in their reasoning, conflicts must be avoided because they are unmanageable. Consequently, communication in HC co-parenting situations generally tends to be absent or pathogenic (Lebow, 2003).

The reasons for disagreements, explicitly expressed by parents, can conceal feelings of injustice linked to the marital breakup. The implicit motivations of the conflict are then of settling scores, of paying a debt to the level of narcissistic reparations. Interparental transactions appear to be influenced by beliefs of injustice. These results encourage reflection regarding intervention paths focused on relational ethics, a concept that takes into consideration issues of justice and injustice (Boszormenyi-Nagy and Spark, 1973). Yet, parents in HC situations want an immediate solution to the discomfort associated with the conflict. In this context, they believe that going to court and letting the judge decide will end the conflict. The parent disengages in this way for the benefit of a judicial decision that will never be truly satisfactory (Mulon, 2011). Feelings of injustice can lead to judicial escalation, and lawyers can help calm the conflict or sharpen it according to their skill and ethics (Le Run, 2012). Therefore, the legal system can exacerbate interparental conflict because it focuses on a win/lose logic, which encourages parents to strengthen this dynamic, by emphasizing blame and reciprocal control strategies (Neff and Cooper, 2004; Martinson, 2010). Consequently, conflict in the litigation process can be fueled by the adversarial nature of the legal system and by the procedures and practices of lawyers. This can exacerbate and perpetuate conflict as each party describes their perspective on the relationship and the causes and consequences of its breakup. Such practices can turn former spouses, who previously got along fairly well, into enemies (Birnbaum and Bala, 2010). For some parents, the triangulation of family courts and other family rights services may be a means by which one or both parents might seek to punish, control, publicly dishonor or condemn the other parent (Francia et al., 2019).

Worsening conflict reduce parents’ ability to manage co-parenting disagreements. Interparental flexibility is also diminished. Often parents in HC co-parenting prefer to maintain unilateral decision making where parental decisions are made by each parent without dialogue, consultation, or interparental negotiation. For them, reciprocal interparental concessions in the name of relational balance do not seem feasible. In HC, co-parenting flexibility and stability become contradictory. For some parents, it is only when things are set in stone that stability is possible. These parents seem to believe that rigidity, manifested through the respect without exception of the pre-established type of childcare, makes it possible to avoid interparental arguments. Sometimes inflexibility becomes a form of interparental revenge for the suffering endured, and parents use the judge’s decisions to justify their inflexibility within the co-parenting relationship. Thus, the judgment becomes instrumentalized for the benefit of personal vengeance.

Our results led us to add a fourth main theme: “Healing the separation.” The absence of this theme in the previous studies (Stolnicu and Hendrick, 2017; Stolnicu, 2020) may be related to the fact that in LC co-parenting situations, separation wounds appear to be, if not “cured,” at least relatively benign. Therefore, it has little or no adverse consequences for former spouses. “Healing the separation” is a process that involves the evacuation of the “emotional overflow,” the identification and work of what “blocks” and the distinction between the marital past and the present and the future co-parent.

Consequently, while this theme is generally absent or blurred in ex-spouses who have experienced a relatively peaceful separation, it becomes significant in ex-spouses who have experienced a conflictual separation. Indeed, it is this second cohort that the professionals work with. This means that, even if the explicit request may only concern children, it is marital wounds that may motivate the consultation. Besides common sense and empathy demand that we not shy away from these problems; they deserve attention because they interfere with parents’ judgment and attitudes toward children. In summary, the development of marital injury, or lack thereof, constitutes the main difference between the parents in LC and HC co-parenting.

Our research shows that in situations of HC co-parenting to heal the separation it is necessary to allow the parents to evacuate their emotional overflow. In addition, allowing the expression of pain, hurt, anger and possible feelings of injustice associated with marital separation can help identify what continues to “block” parents in the conflict.

Through our results we highlighted the importance of understanding the way in which each parent handles separation according to their own psychic economy. As a corollary, in addition to the relational dynamic, elements related to the psychic economy of each parent block collaboration at the level of co-parenting. Possible individual psychopathologies may also play a role in increasing interparental conflict (Neff and Cooper, 2004). Sometimes aspects of divorce can resonate with long-standing vulnerabilities. Indeed, marital separation can be one of the most stressful and vulnerable periods in an adult’s life, thus leading to destructive aggression or rage toward the other parent to mask feelings of sadness and loss (Demby, 2009).

Finances are often another source of conflict. However, Le Run (2012) brings an important nuance to the interpretation of these results by underlining the extremely frequent intermingling of feelings with questions of money which underlie, animate, revive or appease many conflicts. It is about making people pay, both literally and figuratively, for separation, abandonment and betrayal.

Conflict can result from psychological responses to feelings of abandonment, betrayal, anger, hurt or humiliation resulting from the ex-spouse’s conduct or the process of divorce itself (Birnbaum and Bala, 2010). Likewise, interparental financial conflicts can hide pain and feelings of injustice that make co-parenting and dialogue more difficult (Russell et al., 2016). In addition, our results objectify the fact that the co-parenting relationship can be hampered by the difficulties of each parent linked to their own experience of the family past. These results encourage reflection regarding parents’ “construction of the world” (Elkaïm, 2010). In other terms, sometimes individuals’ personal history may present similar painful experiences which tend to be recurrent. This repetition may play an essential role in the interparental conflict by influencing the way parents consider what is happening to them, their expectations, their reactions, their interpretations, and their initiatives. Consequently, these repeated and related wounds may lock parents in a deep belief that will restrict their vision and their freedom of action. Additionally, not feeling recognized by the other parent for their suffering related to past wounds can prevent that parent from investing in improving the quality of the co-parenting relationship. The asynchrony of the mourning process of the marital relationship was also highlighted in our research. Indeed, parents can find themselves in different temporalities in relation to marital separation. The person initiating the separation may have considered marital dissolution for several years previously. As such, the initiator of the separation was able to better detach themself emotionally and give meaning to this event (Bevvino and Sharkin, 2003). Along the same lines, initiators may exhibit more positive emotions and are more likely to be resilient, while non-initiators experience more negative emotions (Frisby et al., 2012).

The ability to forgive, can be seen as a personal strength that leads to improvements in the co-parenting relationship. Therefore, “forgiveness” can represent an individual’s attempt to cope with the painful memory of an event and promotes a reduction in negative responses and an increase in goodwill toward the transgressor (Maltby et al., 2001; Braithwaite et al., 2011). Likewise, personal beliefs and convictions can help parents better manage their conflicts and difficulties related to the separation. Along the same lines, spirituality and religion can support resilience as powerful sources of hope, meaning, peace, solace and forgiveness (Brewer-Smyth and Koenig, 2014).

Regarding clinical interventions in HC co-parenting situation our findings stress the necessity to care about “Healing the separation.” Otherwise, the problems parallel to this axis may completely invade the other three dimensions to the point of obscuring them. Thus, professionals’ availability to respect parent’s temporality and support them through their shared experience is essential.

“Healing the separation” further suggests that the wounds resulting from separation—which may or may not obscure the older wounds related to the couple’s history—are a major barrier to balanced post-separation co-parenting.

These findings contribute in several ways to our understanding of HC co-parenting situations and provide a basis for clinical interventions. Parents “caught” in conflicts may find it difficult to envisage change. Through our results, we emphasize that in order to help parents consider change within their co-parenting relationship, it is needed to identify the barriers that prevent change to happen. Our study shows that the essential elements underlying these parents’ difficulties may reside at a multiplicity of levels: inter-relational, contextual and intrapsychic. Each level contains key potential factors in understanding these families, and in formulating intervention guidelines to improve these problems and encourage change. Difficulties are habitually experienced on various levels at the same time. Identifying what blocks parents in HC situations will then prepare and create beneficial conditions for change.

Our results allow to conceptualize post-divorce co-parenting on a continuum between the HC position and the LC position (Stolnicu and Hendrick, 2017; Stolnicu, 2020). This continuum includes for the four axes, a variety of behaviors and interactions between parents as co-parents, but also between parents, children and other family members. Co-parenting after marital separation is a dynamic process likely to maintain, deteriorate, or evolve depending on a variety of circumstances identified in this study. Indeed, our results highlight characteristics and processes that make it possible to better differentiate between various separation scenarios. This process-based approach does not intent to stigmatize and “change” individuals or families but above all seeks to encourage trajectories’ and interactions’ change and aims to mobilize salutogenic dynamics.

Though, our results enrich the understanding of the emergence and persistence of HC co-parenting. Although post-divorce co-parenting is a well-studied family process, previous literature has not been informed by a conceptual model of co-parenting, and mostly assess co-parenting as an unidimensional construct (Lamela et al., 2016). In the same line with Feinberg (2003) our research conceptualizes co-parenting as a multidimensional construct by nature. Previous literature has attested to the importance of several characteristics of the four co-parenting dimensions evidenced in this study. However, to our knowledge, these characteristics are considered independently without considering their reciprocal interactions and influences. Therefore, the strength of our findings is the integration of all these characteristics into a dynamic and processual conceptualization.

Hence, our results provide pathways which may help professionals distinguish each family’s specific co-parenting difficulties, and therefore adapt their interventions. Different families may manifest HC in different ways. However, our conceptualization allows this phenomenon between families to be identified and distinguished according to each family context. Consequently, as noted by our participants, there is no intervention “recipe.” In other words, specific manifestations of the four axes should be considered in the context of each intervention regarding the particularities of each situation.

Although professionals’ perspective is an important resource which provides access to a variety of experiences regarding HC situations, parents’ and professionals’ perspectives may be quite different (Bertelsen, 2021a,b). Therefore, it would be interesting to deepen the understanding of the studied phenomena by interviewing parents in HC co-parenting situations.

Our conceptualization of HC post-divorce co-parenting is focused on professionals’ experience regarding parents’ sessions. Therefore, intervention pathways target parents’ sessions. Future research should focus on the possible influence of children and/or the extended family. This would allow HC co-parenting interventions to be enriched, and this is the direction that we intend to follow next.

## Data Availability Statement

For confidentiality reasons the interview transcripts for this study will not be made publicly available. They have been deidentified, but the content is still personal and cannot be made fully anonymous. Requests to access the datasets should be directed to AS, alexandra.stolnicu@umons.ac.be.

## Ethics Statement

The studies involving human participants were reviewed and approved by University of Mons. The patients/participants provided their written informed consent to participate in this study.

## Author Contributions

AS: preparation of the theoretical framework, methodological framework, data collection, data analysis, discussion elaboration, and writing the manuscript. SH, JD, and JG: methodological framework supervision, data analysis supervision, and general revision of the manuscript. All authors contributed to the article and approved the submitted version.

## Conflict of Interest

The authors declare that the research was conducted in the absence of any commercial or financial relationships that could be construed as a potential conflict of interest.

## Publisher’s Note

All claims expressed in this article are solely those of the authors and do not necessarily represent those of their affiliated organizations, or those of the publisher, the editors and the reviewers. Any product that may be evaluated in this article, or claim that may be made by its manufacturer, is not guaranteed or endorsed by the publisher.

## References

[ref1] Afifi T. D. Huber F. N. Ohs J. (2006). Parents’ and adolescents’ communication with each other about divorce-related stressors and its impact on their ability to cope positively with the divorce. J. Divorce Remarriage 45, 1–30. doi: 10.1300/J087v45n01_01

[ref2] Anderson S. R. Anderson S. A. Palmer K. L. Mutchler M. S. Baker L. K. (2011). Defining high conflict. Am. J. Fam. Ther. 39, 11–27. doi: 10.1080/01926187.2010.530194

[ref3] Bacon B. L. McKenzie B. (2004). Parent education after separation/divorce. Fam. Court. Rev. 42, 85–98. doi: 10.1177/1531244504421007, PMID: 34148828

[ref4] Baum N. Shnit D. (2003). Divorced parents' conflict management styles. J. Divorce Remarriage 39, 37–58. doi: 10.1300/J087v39n03_02

[ref5] Becher E. H. Kim H. Cronn S. E. Deenanth V. McGuire J. K. McCann E. M.. (2019). Positive parenting and parental conflict: contributions to resilient coparenting during divorce. Fam. Relat. 68, 150–164. doi: 10.1111/fare.12349

[ref6] Beckmeyer J. J. Coleman M. Ganong L. H. (2014). Postdivorce coparenting typologies and children’s adjustment. Fam. Relat. 63, 526–537. doi: 10.1111/fare.12086

[ref7] Bernet W. Wamboldt M. Z. Narrow W. E. (2016). Child affected by parental relationship distress. J. Am. Acad. Child Adolesc. Psychiatry 55, 571–579. doi: 10.1016/j.jaac.2016.04.018, PMID: 27343884

[ref8] Bertelsen B. (2021a). Parent education beyond learning: an ethnographic exploration of a multi-family program for families in post-divorce conflict. Aust. N. Z. J. Fam. Ther. 42, 292–308. doi: 10.1002/anzf.1460

[ref9] Bertelsen B. (2021b). Staying with the conflict—parenting work and the social organization of post-divorce conflict. J. Fam. Stud. 1–17. doi: 10.1080/13229400.2020.1869578

[ref10] Bevvino L. D. Sharkin B. S. (2003). Divorce adjustment as a function of finding meaning and gender differences. J. Divorce Remarriage 39, 81–97. doi: 10.1300/J087v39n03_04

[ref11] Birnbaum R. Bala N. (2010). Towards the differentiation of high-conflict families: an analysis of social science research and Canadian case law. Fam. Court. Rev. 48, 403–416. doi: 10.1111/j.1744-1617.2010.01319.x

[ref12] Boszormenyi-Nagy I. Spark G. M. (1973). Invisible Loyalties: Reciprocity in Intergenerational Family Therapy. New York: Harper and Row

[ref13] Braithwaite S. R. Selby E. A. Fincham F. D. (2011). Forgiveness and relationship satisfaction: mediating mechanisms. J. Fam. Psychol. 25, 551–559. doi: 10.1037/a0024526, PMID: 21707170 PMC3156929

[ref14] Braun V. Clarke V. (2006). Using thematic analysis in psychology. Qual. Res. Psychol. 3, 77–101. doi: 10.1191/1478088706qp063oa

[ref15] Brewer-Smyth K. Koenig H. G. (2014). Could spirituality and religion promote stress resilience in survivors of childhood trauma? Issues Ment. Health Nurs. 35, 251–256. doi: 10.3109/01612840.2013.873101, PMID: 24702209

[ref16] Brown J. H. Bledsoe L. Yankeelov P. Christensen D. Rowan N. L. Cambron M. L. (2009). PACT: a collaborative team model for treating high conflict families in family court. Juv. Fam. Court. J. 60, 49–67. doi: 10.1111/j.1755-6988.2009.01026.x

[ref17] Bullard L. Wachlarowicz M. DeLeeuw J. Snyder J. Low S. Forgatch M.. (2010). Effects of the Oregon model of parent management training (PMTO) on marital adjustment in new stepfamilies: a randomized trial. J. Fam. Psychol. 24, 485–496. doi: 10.1037/a0020267, PMID: 20731495 PMC2928579

[ref18] Cohen O. Finzi-Dottan R. (2012). Defense mechanisms and negotiation as predictors of co-parenting among divorcing couples: a dyadic perspective. J. Soc. Pers. Relat. 30, 430–456. doi: 10.1177/0265407512458657

[ref19] Darwiche J. Nunes C. E. El Ghaziri N. Imesch C. Bessero S. (2021). “Coparenting interventions and shared physical custody: insights and challenges,” in Shared Physical Custody. *Vol. 25*. eds. Bernardi L. Mortelmans D. (Cham: Springer) 25.

[ref20] Demby S. (2009). Interparent hatred and its impact on parenting: assessment in forensic custody evaluations. Psychoanal. Inq. 29, 477–490. doi: 10.1080/07351690903013959

[ref21] Demby S. (2017). Commentary on entrenched postseparation parenting disputes: the role of interparental hatred. Fam. Court. Rev. 55, 417–423. doi: 10.1111/fcre.12286

[ref22] Disner S. G. Beevers C. G. Haigh E. A. P. Beck A. T. (2011). Neural mechanisms of the cognitive model of depression. Nat. Rev. Neurosci. 12, 467–477. doi: 10.1038/nrn3027, PMID: 21731066

[ref23] Donner M. B. (2006). Tearing the child apart. The contribution of narcissism, envy, and perverse modes of thought to child custody wars. Psychoanal. Psychol. 23, 542–553. doi: 10.1037/0736-9735.23.3.542

[ref24] Duerr H. Hautzinger M. (2019). Quantifying the degree of interparental conflict—the spectrum between conflict and forms of maltreatment and abuse. Child Indic. Res. 12, 319–330. doi: 10.1007/s12187-018-9556-1

[ref25] Eikrem T. Jevne K. S. (2022). I do it for the children, and it’s not a walk in the park: Parents’ stories about how to maintain cooperative co-parenting during the divorce process. Child Fam. Soc. Work doi: 10.1111/cfs.12928 [Epub ahead of print].

[ref26] Elkaïm M. (2010). Microtraumatismes, constructions du monde et résilience dans les couples in Famille et Résilience. ed. Delage M. (Odile Jacob), 153–164.

[ref27] Feinberg M. E. (2003). The internal structure and ecological context of Coparenting: a ramework for research and intervention. Parent. Sci. Pract. 3, 95–131. doi: 10.1207/S15327922PAR0302_01, PMID: 21980259 PMC3185375

[ref28] Francia L. Millear P. Sharman R. (2019). Mothers and fathers’ experiences of high conflict past two years post separation: a systematic review of the qualitative literature. J. Child Custody 16, 170–196. doi: 10.1080/15379418.2019.1617821

[ref29] Frisby B. N. Booth-Butterfield M. Dillow M. R. Martin M. M. Weber K. D. (2012). Face and resilience in divorce. J. Soc. Pers. Relat. 29, 715–735. doi: 10.1177/0265407512443452, PMID: 34628664

[ref30] Gulbrandsen W. Haavid H. Tjersland O. A. (2017). High-conflict parents in mediation: an analysis of dialogues and sources to conflict. Conflict Resol. Q. 35, 335–349. doi: 10.1002/crq.21214

[ref31] Houston C. Bala N. Saini M. (2017). Crossover cases of high-conflict families involving child protection services: Ontario research findings and suggestions for good practices. Fam. Court. Rev. 55, 362–374. doi: 10.1111/fcre.12289

[ref32] Karela C. Petrogiannis K. (2018). Risk and resilience factors of divorce and young Children’s emotional well-being in Greece: a correlational study. J. Educ. Dev. Psychol. 8, 68–81. doi: 10.5539/jedp.v8n2p68

[ref33] Kelly J. B. (2012). “Risk and protective factors associated with child and adolescent adjustment following separation and divorce,” in Parenting plan Evaluations: Applied Research for the Family Court. eds. Kuehnle K. Drozd L. (New York, NY: Oxford University Press), 49–84.

[ref34] Lamela D. Figueiredo B. Bastos A. Feinberg M. (2016). Typologies of post-divorce Coparenting and parental well-being, parenting quality and Children’s psychological adjustment, child psychiatry. Hum. Dev. 47, 716–728. doi: 10.1007/s10578-015-0604-5, PMID: 26518292

[ref35] Le Run J. (2012). Les séparations conflictuelles: du conflit parental au conflit de loyauté. Enfanc. Psy 56, 57–69. doi: 10.3917/ep.056.0057

[ref36] Lebow J. (2003). Integrative family therapy for disputes involving child custody and visitation. J. Fam. Psychol. 17, 181–192. doi: 10.1037/0893-3200.17.2.181, PMID: 12828015

[ref37] Maltby J. Macaskill A. Day L. (2001). Failure to forgive self and others: a replication and extension of the relationship between forgiveness, personality, social desirability and general health. Personal. Individ. Differ. 30, 881–885. doi: 10.1016/S0191-8869(00)00080-5

[ref38] Martinson D. J. (2010). One case–one specialized judge: why courts have a duty to manage alienation and other high-conflict cases. Fam. Court. Rev. 48, 180–189. doi: 10.1111/j.1744-1617.2009.01297.x

[ref39] Moral M. A. Chimpén-López C. A. Lyon T. R. Adsuar J. C. (2021). The relationship between differentiation of self and psychological adjustment to separation. Health 9:738. doi: 10.3390/healthcare9060738, PMID: 34208457 PMC8235787

[ref40] Mulon É. (2011). L’enfant dans les séparations conflictuelles: le rôle de la justice. Enfanc. Psy 52, 49–58. doi: 10.3917/ep.052.0049

[ref41] Neff R. Cooper K. (2004). Parental conflict resolution: six-, twelve-, and fifteen-month follow-ups of a high-conflict program. Fam. Court. Rev. 42, 99–114. doi: 10.1177/1531244504421008

[ref42] Petren R. E. Ferraro A. J. Davis T. R. Pasley K. (2017). Factors linked with coparenting support and conflict after divorce. J. Divorce Remarriage 58, 145–160. doi: 10.1080/10502556.2017.1300013

[ref43] Pires M. Martins M. (2021). Parenting styles, coparenting, and early child adjustment in separated families with child physical custody processes ongoing in family court. Child. Aust. 8:629. doi: 10.3390/children8080629, PMID: 34438520 PMC8394593

[ref44] Rød P. A. Iversen A. C. Underlid K. (2013). The child welfare service’s assessments in custody cases that involve minors. Eur. J. Soc. Work. 16, 470–488. doi: 10.1080/13691457.2012.709484

[ref45] Russell L. T. Beckmeyer J. J. Coleman M. Ganong L. (2016). Perceived barriers to postdivorce coparenting: differences between men and women and associations with coparenting behaviors. Fam. Relat. 65, 450–461. doi: 10.1111/fare.12198

[ref46] Smith J. Flowers P. Larkin M. (2009). Interpretative Phenomenological Analysis Theory, Method and Research. London: Sage

[ref47] Smyth B. M. Moloney L. J. (2019). Post-separation parenting disputes and the many faces of high conflict: theory and research. Aust. N. Z. J. Fam. Ther. 40, 74–84. doi: 10.1002/anzf.1346

[ref001] Stokkebekk J. Iversen A. Hollekim R. Ness O. (2021). “The troublesome other and I”: Parallel stories of separated parents in prolonged conflicts. J. Marital Fam. Ther. 47, 52–68. doi: 10.1111/jmft.12474 33421170

[ref48] Stolnicu A. (2020). Rester des parents à vie !? Vers une compréhension processuelle de la coparentalité post séparation conjugale au regard de ses fragilités et de ses ressources. Dissertation. University of Mons, Mons, Belgium

[ref49] Stolnicu A. Hendrick S. (2017). Vers une coparentalité satisfaisante après la séparation conjugale. Thérap. Famil. 38, 415–435. doi: 10.3917/tf.174.0415

[ref50] Taylor R. J. (2001). Listening to the children. J. Divorce Remarriage 35, 147–154. doi: 10.1300/J087v35n01_09

[ref51] Van Lawick J. Visser M. (2015). No kids in the middle: dialogical and creative work with parents and children in the context of high conflict divorces. Aust. N. Z. J. Fam. Ther. 36, 33–50. doi: 10.1002/anzf.1091

[ref52] Yárnoz-Yaben S. Garmendia A. (2016). Parental divorce and emerging adults’ subjective well-being: The role of “carrying messages”. J. Child Fam. Stud. 25, 638–646. doi: 10.1007/s10826-015-0229-0

